# Complex Congenital Heart Diseases and Pregnancy: Maternal and Fetal Risks

**DOI:** 10.36660/abc.20190764

**Published:** 2019-12

**Authors:** Valéria de Melo Moreira

**Affiliations:** Universidade de São Paulo - Faculdade de Medicina dos Hospital das Clinicas do Instituto do Coração, São Paulo, SP - Brazil

**Keywords:** Pregnancy/complications, Heart Defects,Congenital/ complications, Heart Defects,Congenital/trends, Maternal Mortality, Fetal Mortality, Maternal and Fetal Procedure Outcomes.

The diagnosis and treatment of congenital heart disease has made remarkable progress in recent decades and the success of this approach is evidenced by the increasing number of adults with congenital heart disease. Many of these patients have residual injuries or have undergone palliative surgery and face additional challenges in adulthood, requiring integrated care to reach a full life potential.^1^

Consequently, the number of women of childbearing age with congenital disease submitted to surgical repair, palliative procedure or the natural evolution of the disease is increasing, with a higher maternal and fetal risk in the presence of heart disease. Reproductive counseling is essential, by informing the consequences and possible complications and advising against pregnancy in the presence of more complex defects.^2^

There are several classification models and risk predictors applied to maternal cardiovascular involvement that helP in the counseling and clinical management of these patients.^3^ Pijuan-Domenech et al.^4^ demonstrated that the modified version of the classification devised by the World Health Organization (WHO) is the best predictor of cardiac complications in pregnancy compared to other risk prediction models and it is the best accepted model for pregnancy in women with congenital heart disease.^4^

Pregnancy brings profound hemodynamic alterations and these physiological adaptations occur to allow an adequate adjustment of the metabolic needs of the mother and fetus, providing an adequate placental perfusion. Heart rate rises by 15 to 30%, peaking at the end of the second or the start of the third trimester. There is an increase in preload due to an increase in plasma volume and cardiac output increases by 30 to 50%. Additionally, there is an increase in the endothelial production of prostacyclin and nitric oxide, promoting a reduction in total vascular resistance.

Such cardiovascular adjustments are easily supported by women with normal cardiac reserve. However, these alterations may not be well tolerated in pregnant women with congenital heart disease, especially those with complex heart disease and limited capacity to adapt to significant hemodynamic alterations, which may lead to decompensation and increased risk of adverse maternal-fetal outcomes.^5^

Patients with complex structural alterations are associated with a higher chance of arrhythmias, decompensated heart failure and thromboembolic events. The volume overload that occurs in pregnancy, associated with increased excitability of adrenergic receptors caused by hormonal factors may facilitate the development of arrhythmias in patients with structural or residual cardiac defects after repair. The formation of scar tissue on the site of surgical manipulation may be involved with one of the pathophysiological factors of arrhythmias.^6^ The heterogeneity and complexity of these malformations require specific management strategies and a multidisciplinary approach.^7^

The fetal and neonatal outcomes are also closely related to the complexity and severity of the maternal congenital heart disease. Early gestational losses and intrauterine growth restriction have been reported in these pregnancies.^8^

In this issue of the *Arquivos Brasileiros de Cardiologia*, Avila et al.,^9^ evaluated the evolution of pregnancy in patients with complex congenital heart disease, in an attempt to identify variables that could lead to a higher risk of unfavorable maternal-fetal outcome. A retrospective and observational study covering the last ten years, carried out in a single Cardiology and Obstetrics center, included 42 pregnancies in 40 patients with complex congenital heart disease classified as risk category III by the WHO, which means the woman is advised to avoid pregnancy.

The study results are in accordance with the worldwide literature, which shows a high rate of maternal and fetal problems. The main complications were heart failure and arrhythmia, and there were two maternal deaths due to obstetric causes. Among the most frequent structural defects were the transposition of the great arteries (with repair at the atrial or arterial level) and univentricular heart (Fontan procedure).

In the study, most patients with transposition of the great arteries had favorable maternal and fetal evolution. It is known that in patients with transposition of the great arteries, the risks associated with pregnancy are mainly related to patients submitted to repair at atrial level (Senning and Mustard procedure). There is a higher risk of developing arrhythmias and ventricular dysfunction of the systemic ventricle. Regarding patients submitted to arterial exchange (Jatene procedure), although the risk seems to be lower, greater attention should be paid to cases of dilated neoaorta or other residual complications.^10^

As for the univentricular grouP , in late postoperative period after Fontan surgery, an incapability to adapt to the pregnant condition was observed, with decompensation and functional worsening in all patients. It should be noted that in the hypoxemic scenario, the risk of poor maternal and fetal evolution is considerably present.

This issue is very relevant, since complex congenital heart disease has a very heterogeneous spectrum and few studies have evaluated whether maternal and fetal outcomes differ between the subtypes of this subgroup. More targeted studies may provide more accurate information for the counseling and better management of these patients.
